# Temporal-lobe functional connectivity in future converters to Alzheimer’s disease

**DOI:** 10.1192/j.eurpsy.2025.1691

**Published:** 2025-08-26

**Authors:** Y. R. Panikratova, A. Y. Komarova, E. G. Abdullina, O. V. Bozhko, N. S. Cherkasov, I. V. Kolykhalov, I. S. Lebedeva

**Affiliations:** 1 Laboratory of neuroimaging and multimodal analysis; 2 Department of radiology; 3 Department of gerontological psychiatry, Mental Health Research Center, Moscow, Russian Federation

## Abstract

**Introduction:**

Preclinical stages of Alzheimer’s disease (AD) are characterized by structural and neurochemical abnormalities in temporal lobe and temporoparietal junction (T-TPJ; Popuri *et al.* Hum Brain Mapp 2020; 41 4127-4147; Jagust. Nat Rev Neurosci 2018; 19 687-700). However, the role of functional characteristics of T-TPJ in conversion to AD is understudied.

**Objectives:**

Our aim was to clarify whether any patterns of functional connectivity (FC) within T-TPJ differentiate patients with amnestic mild cognitive impairment (aMCI) and future conversion to AD from stable aMCI and healthy controls.

**Methods:**

Patients with aMCI and future conversion to dementia due to AD (converters, *n* = 15; mean age 74.31 ± 7.86), patients with stable aMCI (non-converters, *n* = 12; mean age 66.77 ± 9.54), and healthy individuals without cognitive deficits (*n* = 29; mean age 64.17 ± 11.30) underwent resting-state fMRI (3T Philips Ingenia scanner). Eighteen T-TPJ cortical ROIs in each hemisphere were defined according to Harvard-Oxford atlas (Desikan *et al.* Neuroimage 2006; 31 968-980). FC between these ROIs was compared between groups separately for each hemisphere (one-way ANCOVA with connection threshold *p[FDR]* < 0.05 and *post hoc* between-group comparisons). Age, sex, and number of outlier scans due to motion were included into the models as covariates of no interest. The analyses were implemented via CONN (RRID:SCR_009550; www.nitrc.org/projects/conn).

**Results:**

FC between the posterior parts of left middle and inferior temporal gyri was different between converters, non-converters, and healthy individuals (*p[FDR]* = .0256). Converters demonstrated an increased FC between these regions compared to other groups (*p* = .0003 for both tests). We also observed a trend for inverse correlation between this FC and delayed recall of words (MoCA) in the entire sample (*p* = .055).

**Conclusions:**

An increased FC between the temporal brain regions may reflect either pathological processes that have already started, or compensatory mechanisms, or both in future converters to AD. The posterior part of left middle temporal gyrus is critical for auditory verbal memory, whereas the posterior inferior temporal cortex in the left hemisphere stores visual images associated with a word. The observed trend-like correlation might indicate that patients with worse auditory verbal memory rely on visual images associated with a word, however, this assumption should be supported in further studies.

The study was supported by RSF grant 24-15-00220.

**Disclosure of Interest:**

None Declared

